# Impact of Forest Harvesting on Trophic Structure of Eastern Canadian Boreal Shield Lakes: Insights from Stable Isotope Analyses

**DOI:** 10.1371/journal.pone.0096143

**Published:** 2014-04-24

**Authors:** Patricia Glaz, Pascal Sirois, Philippe Archambault, Christian Nozais

**Affiliations:** 1 Département de biologie et centre d’études nordiques, Université du Québec à Rimouski, Rimouski, Québec, Canada; 2 Chaire de recherche sur les espèces aquatiques exploitées, Laboratoire des sciences aquatiques, Département des sciences fondamentales, Université du Québec à Chicoutimi, Chicoutimi, Québec, Canada; 3 Institut des sciences de la mer de Rimouski, Université du Québec à Rimouski, Rimouski, Québec, Canada; University of Shiga Prefecture, Japan

## Abstract

Perturbations on ecosystems can have profound immediate effects and can, accordingly, greatly alter the natural community. Land-use such as forestry activities in the Canadian Boreal region have increased in the last decades, raising concerns about their potential impact on aquatic ecosystems. The objective of this study was to evaluate the impact of forest harvesting on trophic structure in eastern Canadian Boreal Shield lakes. We measured carbon and nitrogen stable isotopes values for aquatic primary producers, terrestrial detritus, benthic macroinvertebrates, zooplankton and brook trout (*Salvelinus fontinalis*) over a three-year period in eight eastern Boreal Shield lakes. Four lakes were studied before, one and two years after forest harvesting (perturbed lakes) and compared with four undisturbed reference lakes (unperturbed lakes) sampled at the same time. Stable isotope mixing models showed leaf-litter to be the main food source for benthic primary consumers in both perturbed and unperturbed lakes, suggesting no logging impact on allochthonous subsidies to the littoral food web. Brook trout derived their food mainly from benthic predatory macroinvertebrates in unperturbed lakes. However, in perturbed lakes one year after harvesting, zooplankton appeared to be the main contributor to brook trout diet. This change in brook trout diet was mitigated two years after harvesting. Size-related diet shift were also observed for brook trout, indicating a diet shift related to size. Our study suggests that carbon from terrestrial habitat may be a significant contribution to the food web of oligotrophic Canadian Boreal Shield lakes. Forest harvesting did not have an impact on the diet of benthic primary consumers. On the other hand, brook trout diet composition was affected by logging with greater zooplankton contribution in perturbed lakes, possibly induced by darker-colored environment in these lakes one year after logging.

## Introduction

Every ecosystem is susceptible to perturbations that can alter its structure and function [1], [2]. Perturbations include fires, flooding, windstorms, insect outbreaks, as well as anthropogenic disturbances such as forest harvesting [3]. In the Canadian Boreal ecoregion, forest harvesting has increased in the last decades, raising concerns about its potential impact on natural biogeochemical processes in soils and the export pathways that deliver dissolved nutrients and organic matter to aquatic ecosystems. Indeed, forests adjacent to aquatic systems can strongly influence aquatic community as they provide a considerable amount of organic matter [4]. Recent studies have demonstrated that organic matter from riparian forest (allochthonous matter) is an important food resource for invertebrate consumers [5]–[7] and fish [6]–[9] in unperturbed lakes.

Disturbances to forest watersheds by forest management activities have been better documented in lotic than in lentic ecosystems [10]–[13]. However, studies in the Boreal Shield ecozone have reported that forest harvesting in lake catchments can modify water quality. For instance, increases in total phosphorous (TP) and dissolved organic carbon (DOC) concentrations were reported after logging activities [14]–[16]. Higher chlorophyll *a* (chl *a*) concentrations have also been observed in logged compared to unlogged lakes [16], [17]. Recently, [16] showed that changes in water quality and zooplankton community structure due to forestry activities in eastern Canadian Boreal Shield lakes influenced the feeding success of age-0 yellow perch (*Perca flavescens*). Also, brook trout *(Salvelinus fontinalis)* catch per unit of effort (CPUE) and biomass per unit of effort (BPUE) decreased, respectively, by 18% and 22% in lakes after clear-cutting [18]. These changes reflected a significant modification in population dynamics probably caused by logging operations [18].

*S. fontinalis* is a widely distributed fish species in eastern Canadian Boreal Shield lakes and is an economically important game fish in Quebec [19]. It has been identified as a generalist carnivore fish that feeds mainly on benthic macroinvertebrates, as revealed by stable isotope analyses [7] and stomach content data [20], [21]. However, it can also feed on zooplankton [7], terrestrial insects [22], [23] and fish, including brook trout [21], [24]. Previous studies have shown that stable isotope signatures could be used to identify intraspecific shifts in feeding strategies of brook trout [22], [23]. However, little is known about logging impact on brook trout diet and on its size-related diet shifts in Canadian Boreal Shield lakes. A change in its diet towards zooplanktivory was observed in logged lakes in Quebec based on stomach content analyses [23]. Since forest harvesting is likely to affect brook trout habitat, more information is needed to assess the impact of forest harvesting on trophic structure in Canadian Boreal Shield lakes.

In this study we measured naturally occurring carbon and nitrogen stable isotope ratios of basal resources (terrestrial detritus, macrophytes, periphyton and sediment organic matter), benthic organisms, zooplankton, and brook trout in eight oligotrophic Canadian Boreal Shield lakes both before and after logging. Using stable isotope analysis, we examined feeding relationships among consumers to investigate the impact of forest harvesting on primary consumers and brook trout diet. We also examined size-related diet shifts of brook trout in perturbed and unperturbed lakes.

## Methods

### Ethic Statement

This study was carried out in accordance with current laws and ethical concerns in Québec, Canada, being reviewed and approved by the Ministère des Ressources Naturelles et de la Faune (Permit numbers: 2008: MRNF-2008070986802SP; 2009: MRNF-2009071392402SP; 2010: MRNF-2010070798002SP). The research involved no endangered and protected species. The animal use protocol for this research was reviewed and approved by the Comité de protection des animaux at the Université du Québec à Rimouski (CPA-UQAR) (Certification number: CPA-UQAR-27-07-51) and by the Comité de protection des animaux at the Université du Québec à Chicoutimi (CPA-UQAC) (Certification number: CPA-UQAC-R06-10).

### Study Area

**Figure 1:**
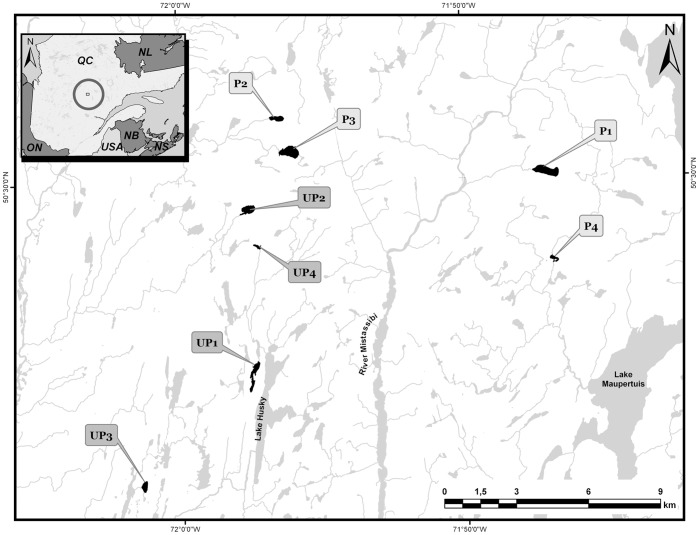
Location of the eight study lakes sampled in 2008, 2009 and 2010. UP, unperturbed lakes; P, perturbed lakes (harvested in 2009).

The study was conducted in the province of Québec, on the Boreal Shield, on the Mistassibi River drainage basin (50° 7'30' N, 71° 35'59' W) (Fig. 1). The boreal forest of the study area is mainly dominated by virgin black spruces *(Picea mariana)*. The soil layer over the rock is thin and lakes in this region are oligotrophic [15].

### Sampling

Eight lakes with similar morphometric characteristics were selected for this study [7]. These eight lakes were unperturbed in 2008 at the beginning of this survey. In 2009 and 2010, the drainage basin of four of these lakes were kept undisturbed (unperturbed lakes) and four other lakes (perturbed lakes) where harvested about 70% of lake catchment during autumn 2008 (Fig. 1). All lakes were sampled once in July 2008, 2009 and 2010. Forest was cut using the careful logging around advanced growth (CLAAG) strategy which preserves advanced growth on site after harvesting. The advanced growth contributes to site stocking as well as a seed source (larger trees) to regenerate open areas. Under this treatment, all trees equal to or greater than 10 cm diameter at breast height (d.b.h.) are harvested [25]. All lakes have a drainage ratio higher than 4 and perturbed lakes had a catchment area cut between 69–77%. A 20 m strip of standing forest was intentionally kept along lakes after harvesting activities. In all lakes, the only fish species found was brook trout (*Salvelinus fontinalis*) except for perturbed lake 4, in which brook trout coexists with white sucker (*Catostomus commersoni*) (Fig. 1). In each lake, five littoral stations were set uniformly around the lake. Water samples (4L) were collected at each of these stations at 50 cm below the surface with an Alpha bottle for the determination of DOC, TP, Chl *a* concentrations and isotopic analysis of particulate organic matter (POM). Water samples for Chl *a* and POM analysis were filtered onto Whatman GF/F filters and kept frozen at −20°C until analysis. Samples for DOC measurements were maintained at 4°C, whereas TP samples were kept frozen at −20°C until analysis.

Terrestrial detritus (leaf litter, woody debris), macrophytes, periphyton (epilithon- rock biofilms, and epixylon-wood biofilms), benthic macroinvertebrates, zooplankton, and brook trout were sampled at each station for stable isotope analyses. Terrestrial detritus, macrophytes and periphyton were hand-collected while benthic macroinvertebrates were sampled using a Turtox D-net with a mesh size of 500 µm, and stored in cooler boxes. Zooplankton samples were obtained by towing for 5–10 min a plankton net (mouth opening 20 cm, mesh 53µm) mounted alongside a boat. Zooplankton was then placed in Ziploc bags containing water. Fish were captured with six experimental gill nets (mesh sizes of 1″, 1½”, 2″, 2½” 3″, 3½”) set simultaneously around the lake, perpendicular to the shore with the small mesh always set toward the shore, according to Ministry of Sustainable Development, Environment, Wildlife and Parks standard protocol. Gill nets were left over night for 12 hours. Fish samples were placed in cooler boxes. All samples were transported to the laboratory (about 30 minutes from the lakes) for processing.

### Sample Preparation and Analyses

TP was measured using the molybdenum blue method [26] after autoclaving 50 ml samples with 0.5 g of potassium persulfate for 1 h at 120°C. TP was afterwards assessed using an AutoAnalyzer (AA3, Bran+Luebbe, German). For the determination of Chl *a,* pigments were extracted for 24 h in 90% acetone, at 5°C in the dark without grinding. Chl *a* was determined using [27] method. DOC was determined by filtering subsamples of water through precombusted (500°C, 5 h) Whatman GF/F filters. The filtrate was placed in glass vials with teflon-lined caps and acidified with 25% v/v H_3_PO_4_ (10 µL mL^−1^). DOC values were obtained using a TOC-5000A analyzer (Shimadzu, Kyoto, Japan), following the protocol of Whitehead et al. (2000). DOC reference standards were produced by the Hansell’s Certified Reference Materials (CRM) program. Terrestrial detritus, macrophytes and periphyton samples were cleaned with the aid of a dissecting microscope and epibionts were removed. Benthic macroinvertebrates and zooplankton samples were identified at the order or family level using [28] and [29] respectively. After identification, benthic macroinvertebrates were frozen separately at −20°C while zooplankton was frozen in mixed samples at −20°C. Total length of each brook trout was recorded. A 2 cm square and 1 cm deep block of dorsal white muscle without skin sample was taken from 25 brook trouts in each lake. All samples were kept frozen at −20°C in scintillation vials until analysis.

### Stable Isotope Analyses

POM filters, terrestrial detritus, macrophytes, periphyton, benthic macroinvertebrates, zooplankton and brook trout samples were dried at 60°C for 48 h. Mortar and pestle were thereafter used to grind samples into a fine powder (excepting for POM filters). Powder and filter samples were then encapsulated in pressed tin capsules (5×9 mm) and tin foil cups (Costech Analytical Technology), respectively. Encapsulated dry mass was approximately 1 mg for all samples. Zooplankton were pooled together (approximately 50 individuals) to make up 1 mg of sample tissue. Benthic macroinvertebrates were also pooled together when necessary (between 3 and 4 individuals) to achieve 1 mg of sample tissue.

Analyses of stable isotopes ratios of C (δ^13^C) and N (δ^15^N) were carried out at the Institut des sciences de la mer (ISMER, Rimouski, Quebec, Canada) using a COSTECH ECS 4010 Elemental Analyser coupled with a DeltaPlus XP Isotope Ratio Mass Spectrometer (IRMS, Thermo Electron Co). System control as well as acquisition and treatment of the data were carried out using the Isodat 2 software. Stable isotope ratios were expressed in δ notation as parts per thousand (‰) according to the equation:
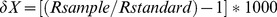
(1)where X is ^13^C or ^15^N and R is the corresponding ^13^C/^12^C or ^15^N/^14^N ratios.

International standards used for the measurement were Vienna Pee Dee Belemite (VPDB) limestone for ^13^C and atmospheric nitrogen for ^15^N. Regional standards for in-lab normalization regressions to determine sample delta values were anhydrous caffeine (Sigma Chemical Co., St-Louis, USA), Mueller Hinton Broth (Becton Dickinson, USA) and *Nannochloropsis*. These homemade standards were calibrated once a year using standards from the National Institute of Standards and Technology (NIST Standard Reference Materials: IAEA-N1, IAEA-N2, IAEA-N3, USGS-40, and USGS-41).. Replicate analyses of standards gave analytical errors (SD) of ±0.30 ‰ for C and ±0.18 ‰ for N.

A mathematical normalization technique was used to standardize lipid content on δ^13^C [30], [31]. We used the mathematical equations proposed by [31], which provides a reliable method for normalizing estimates of δ^13^C for lipid concentration. For aquatic organisms the equation is:
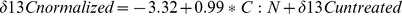
(2)where C:N is the carbon-to-nitrogen ratio of a sample.

The resulting δ^13^C_normalized_ provides an estimate of δ^13^C that is normalized for the effects of lipid concentration on δ^13^C and is comparable to the δ^13^C after direct chemical lipid extraction [31]. Lipid effects were removed for fish samples, as it has been shown by many authors that there is a clear dependence of the δ^13^C difference between lipid-extracted and untreated samples on the C:N ratio [31]–[33]. However, no such relationship was shown for invertebrate and plants [31], [32].

### Data Analyses

To estimate the relative contribution of the different food sources to the diet of brook trout, we adopted a Bayesian multi-source stable isotope mixing model, available as an open source R package (SIAR) [34]. SIAR takes data on organism isotopes and fits a Bayesian model to their dietary habits based upon a Gaussian likelihood with a Dirichlet distribution prior mixture on the mean. SIAR offers a number of advantages over earlier mixing models: it can incorporate trophic enrichment factors within the model and known C and N concentrations dependence, assuming that for each element, a source's contribution is proportional to the contributed mass times the elemental concentration in that source. We assumed a trophic enrichment of +1‰ for ^13^C [35] and +3.4‰ for ^15^N [36]. Brook trout carbon and nitrogen isotope values were compared using two-way analyses of variance (ANOVAs). Factors in the model were: treatment (fixed with two levels, unperturbed and perturbed) and year (fixed with three years of sampling) and their interactions. The same ANOVA model was used to compare TP, Chl *a* and DOC concentrations in all years and treatments. The principal source of variation of interest for impact assessment was the interaction between the treatment (perturbed/unperturbed) and the year. Normality and homogeneity of variances were tested visually in all cases [37]. TP, Chl *a* and DOC variables were log transformed to meet ANOVA assumptions.

Stable isotopes of carbon and nitrogen were plotted against fish total length in each treatment and year. Simple linear regression was calculated for each treatment in the three years and analyses of covariance (ANCOVAs) were used to compare regression lines in each year, with treatment as a factor. Normality and homogeneity of variances were tested visually in all cases [37].

## Results

TP concentrations ranged from 4.80 (±0.12) (perturbed, 2008) to 5.75 (±0.16) µg l^−1^ (perturbed, 2009) (Table 1). A statistical significant interaction between treatment and year was observed for TP concentrations (SS = 0.1473, F_2,6_ = 6.7476, *p* = 0.0108). A posteriori Tukey’s test confirmed that unperturbed and perturbed lakes were significantly different in 2009 (after forest harvesting). Chl *a* values ranged from 0.41 (±0.03) (unperturbed, 2008) to 1.00 (±0.08) µg l^−1^ (perturbed, 2009) (Table 1). Chl *a* values did not show significant differences between treatment and year (SS = 0.2903, F_2,6_ = 1.2495, *p* = 0.3214). DOC concentrations ranged from 11.34 (±0.46) (perturbed, 2008) to 15.27 (±0.32) mg l^−1^ (perturbed, 2009) (Table 1). A statistical significant interaction between treatment and year was observed for DOC concentrations (SS = 32.3253, F_2,6_ = 6.2160, *p* = 0.0466). A posteriori Tukey’s test confirmed that unperturbed and perturbed lakes were not significantly different in 2008 (before forest harvesting) nor in 2010 but they were significantly different in 2009 (after forest harvesting).

**Table 1:** Mean values (±SE) of TP (µg l^−1^), Chl *a* (µg l^−1^) and DOC (mg l^−1^) concentrations in unperturbed (UP) and perturbed (P) lakes in 2008, 2009 and 2010.

Tr	Year	n	TP	Chl a	DOC
UP	2008	4	5.14 (0.17)	0.41 (0.02)	12.04 (0.17)
P	2008	4	4.83 (0.12)	0.65 (0.06)	11.37 (0.52)
UP	2009	4	5.04 (0.16)	0.64 (0.05)	13.13 (0.18)
P	2009	4	5.76 (0.17)	1.00 (0.06)	15.27 (0.35)
UP	2010	4	5.68 (0.11)	0.65 (0.05)	13.26 (0.55)
P	2010	4	5.71 (0.13)	0.66 (0.06)	12.74 (0.35)

**Figure 2:**
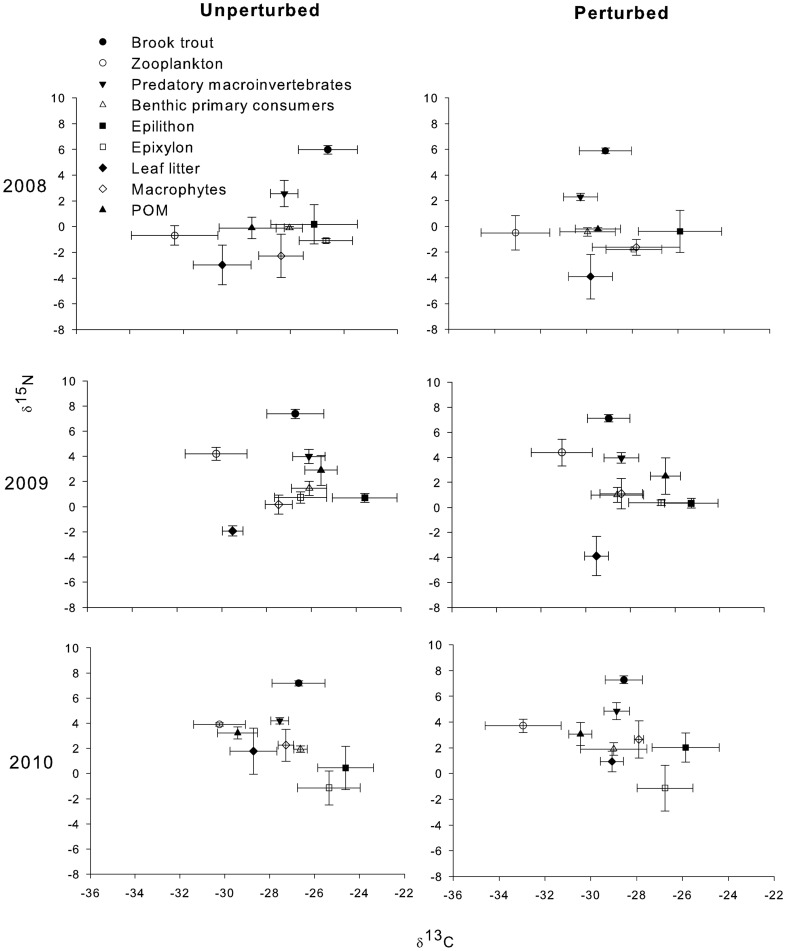
Mean δ^13^C and δ^15^N of potential food sources and brook trout for all years in unperturbed and perturbed lakes. Error bars represent the mean ± SE. δ^13^C and δ^15^N data are expressed in ‰.

Brook trout had higher δ^15^N values in all years and in both unperturbed and perturbed lakes than other consumers, indicating a higher trophic level (Fig. 2). Mayflies (Ephemeroptera), corixids (Corixidae), amphipods (Amphipoda) and caddisflies (Trichoptera), all of them non-predatory macroinvertebrates, were grouped as benthic primary consumers. Leeches (Hirudinea), water mites (Hydracarina), dragonflies (Anisoptera), damselflies (Zygoptera), dysticids (Dysticidae) and alderflies (Sialidae), all of them active predators, Empidids (Empididae) and Chironomids (Chironomidae) which are mainly predators, were pooled as predatory macroinvertebrates. *Daphnia* spp., calanoids (Calanoida) and cyclopoids (Cyclopoida) were pooled as zooplankton. Predatory macroinvertebrates had higher δ^15^N values than zooplankton and benthic primary consumers in 2008 and 2010 in both treatments. However, in 2009 zooplankton δ^15^N values were higher than predatory macroinvertebrates in both unperturbed and perturbed lakes (Fig. 2). Zooplankton had the lowest δ^13^C values in all lakes and treatments (Fig. 2).

A mixing model was performed to evaluate carbon contribution to benthic primary consumers for each year and treatment (unperturbed; perturbed). Benthic primary consumers derive their carbon mainly from leaf litter in all lakes (Table 2). Periphyton (unperturbed 2009, unperturbed 2010) and macrophytes (unperturbed 2008, perturbed 2008, perturbed 2009) also contributed to the diet of benthic primary consumers, with mean contributions of 13, 30, 31, 14 and 20%, respectively. The contribution of these sources was however lower than contribution of leaf litter in all treatments and years (Table 2).

**Table 2:** Bayesian mixing model (SIAR) results for primary consumers in 2008, 2009 and 2010 for unperturbed (UP) and perturbed (P) lakes.

Tr	Year	Leaf litter	Periphyton	Macrophytes	POM
UP	2008	**63; 51–76**	3; 0–6	**31; 15–47**	3; 0–5
P	2008	**76; 69–84**	3; 0–6	**14; 1–25**	7; 0–17
UP	2009	**80; 66–91**	**13; 2–34**	6; 0–13	1; 0–4
P	2009	**69; 59–80**	7; 0–16	**20; 6–35**	4; 0–10
UP	2010	**60; 45–73**	**30; 6–45**	1; 0–4	9; 0–20
P	2010	**87; 69–98**	5; 0–11	1; 0–5	7; 0–23

Values represent the mean and the 1^st^ to 99^th^ percentile range of potential contribution. Sources with a necessary contribution (minimal potential contribution >0%) are in bold.

A mixing model performed on each year and treatment confirmed that brook trout derived its carbon biomass mainly from predatory benthic macroinvertebrates in all lakes, except in perturbed lakes in 2009 (Table 3). In 2009, in perturbed lakes, zooplankton represented a major source of carbon for brook trout, with a mean contribution of 59%, while predatory macroinvertebrates contributed to only 26% to its diet (Table 3). Zooplankton also appeared to make a contribution to brook trout diet in unperturbed lakes in 2009 and 2010 and in perturbed lakes in 2010 but in all these cases, the contribution of predatory macroinvertebrates was higher than the contribution of zooplankton (Table 3). The mixing model also revealed benthic primary consumers as a food source for brook trout in perturbed lakes 2009, 2010 and in unperturbed lakes in 2008 and 2010, although with lower values (15, 23, 13 and 18% respectively) than predatory macroinvertebrates and zooplankton (Table 3). ANOVA’s test showed no significant interaction between treatment and year for brook trout δ^15^N and δ^13^C values (Table 4).

**Table 3:** Bayesian mixing model (SIAR) results for brook trout in 2008, 2009 and 2010 for unperturbed (UP) and perturbed (P) lakes.

Treatment	Year	Zooplankton	Benthic primary consumers	Predatory macroinvertebrates
UP	2008	1; 0–3	**13; 6–2**	**86; 79–92**
P	2008	1; 0–2	1; 0–3	**98; 96–99**
UP	2009	**43; 32–53**	3; 0–7	**54; 42–65**
P	2009	**59; 38–70**	**15; 9–19**	**26; 15–38**
UP	2010	7; 0–15	**18; 12–33**	**75; 65–85**
P	2010	**17; 12–22**	**23; 18–28**	**60; 55–65**

Values represent the mean and the 1^st^ to 99^th^ percentile range of potential contribution. Sources with a necessary contribution (minimal potential contribution >0%) are in bold.

**Table 4:** Results of the two-way ANOVA testing the effect of treatment (tr : perturbed, unperturbed), year and their interactions on δ^15^N and δ^13^C brook trout muscle.

Variable		tr	year	tr x year	Residual
	df	1	2	2	18
δ^13^C	SS	28.4272	0.4088	0.3766	21.1757
	F	24.1641	0.1737	0.1601	
	p	0.0001 (*)	0.8419	0.8533	
δ^15^N	SS	0.0513	9.1760	0.1435	1.4651
	F	0.6333	56.6419	0.8858	
	p	0.4375	<0.0001 (*)	0.4314	

The principal source of variation of interest is the interaction between the treatment and the year of sampling. (*) p<0.05.

**Figure 3:**
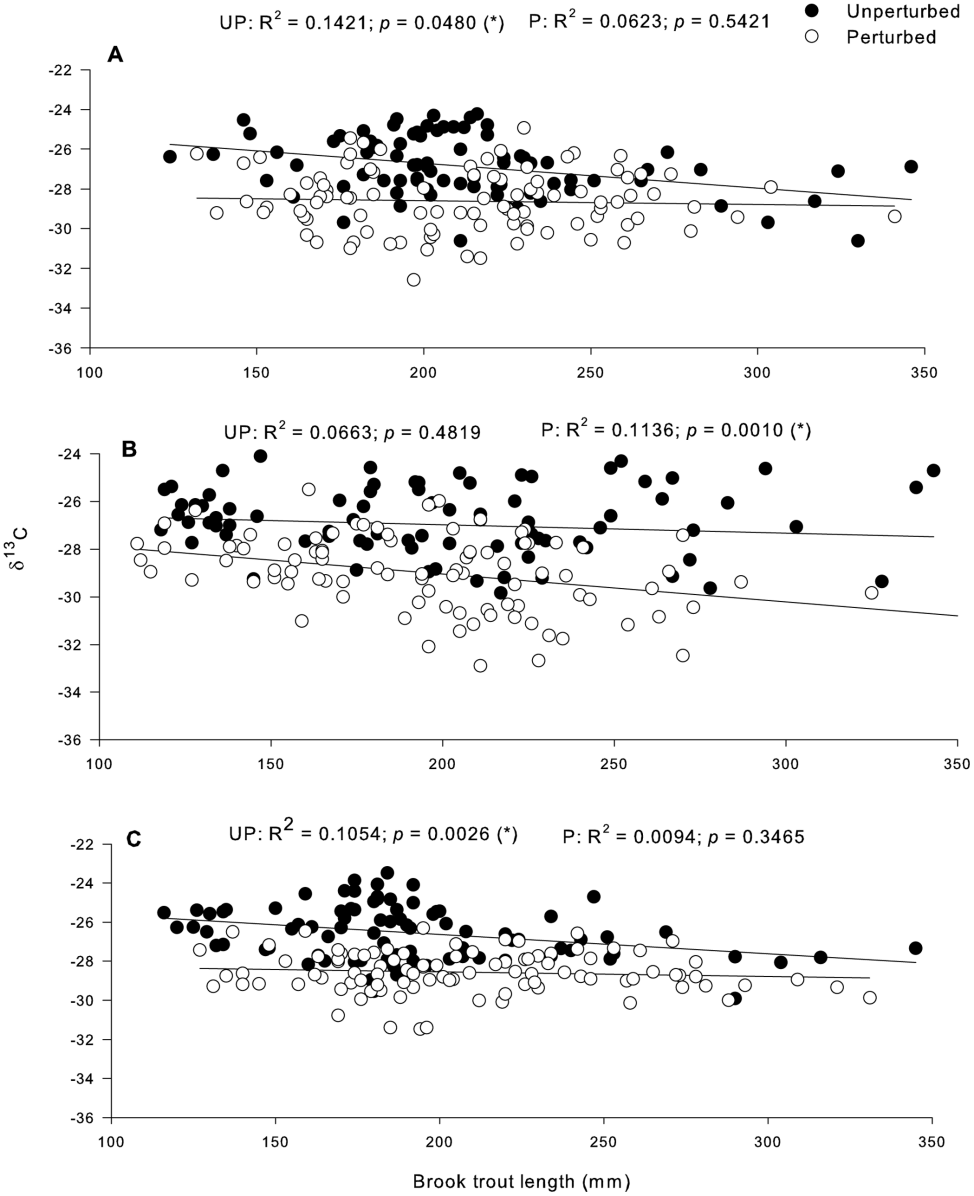
Changes in brook trout δ^13^C signatures with increasing fish length in (a) 2008, (b) 2009 and (c) 2010 in unperturbed and perturbed lakes. δ^13^C data are expressed in ‰. (*) p<0.05.

**Figure 4:**
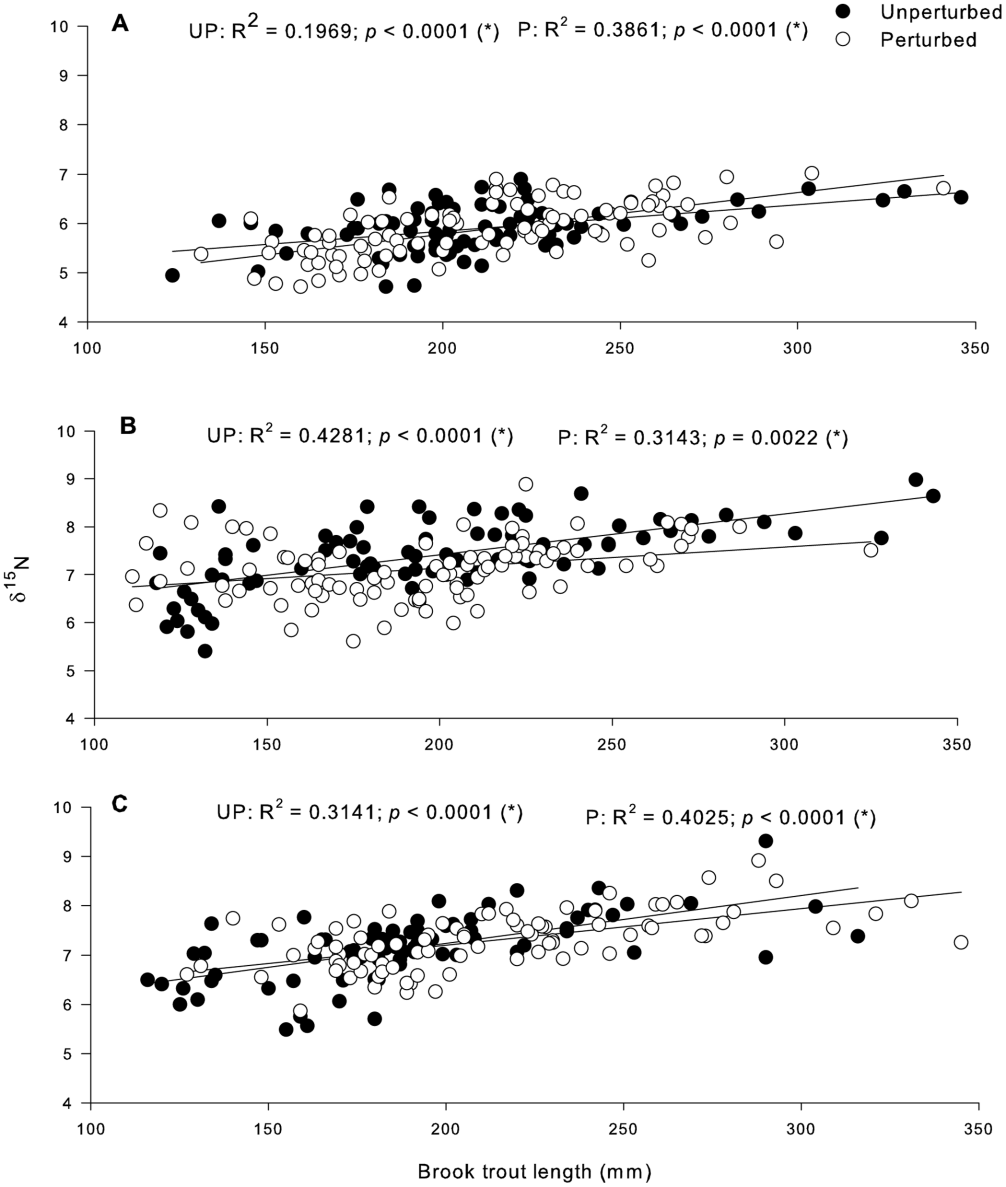
Changes in brook trout δ^15^N signatures with increasing fish length in (a) 2008, (b) 2009 and (c) 2010 in unperturbed and perturbed lakes. δ^15^N data are expressed in ‰. (*) p<0.05.

Brook trout showed great variability in δ^13^C values and it became significantly more depleted in ^13^C with increasing brook trout total length in unperturbed lakes in 2008 and 2010, and in perturbed lakes in 2009. In unperturbed lakes in 2009, and in perturbed lakes in 2008 and 2010, no obvious patterns of δ^13^C values were found in relation to increasing fish length (Fig. 3). Brook trout δ^13^C tended to be more ^13^C-depleted in perturbed lakes than in unperturbed lakes in 2008, 2009 and 2010 (Fig. 3a, 3b and 3c respectively). ANCOVA results for δ^13^C values vs. brook trout total length comparing treatment simple regressions in all years confirmed a significant difference between unperturbed and perturbed lakes in 2009 (F_2,6_ = 4.218, *p* = 0.033) and 2010 (F_2,6_ = 4.222, *p* = 0.040). Brook trout became significantly more enriched in ^15^N with increasing fish length in all cases (Fig. 4). Total length explained from 19% (2008) to 42% of the variation in δ^15^N in unperturbed lakes and from 31% (2009) to 40% (2010) in perturbed lakes (Fig. 4). No significant differences were found between treatments for any year using ANCOVA for δ^15^N values.

## Discussion

No logging impact was observed on the contribution of allochthonous material to the littoral food web since diet of primary consumers has not changed after harvesting. Brook trout derived their food mainly from benthic predatory macroinvertebrates in unperturbed lakes in all years, although in 2009 zooplankton also contributed to its diet (43%). However, this contribution represents a lower proportion than the contribution of benthic predatory macroinvertebrates in 2009 (54%) in unperturbed lakes. Yet, in perturbed lakes in 2009, zooplankton appeared to be the main contributor to brook trout diet. This change in brook trout diet was mitigated two years after the perturbation since in 2010 benthic predatory macroinvertebrates was the main carbon source for brook trout in both unperturbed and perturbed lakes. Size-related diet shift was also observed for brook trout as it became significantly more ^13^C-depleted with increasing fish length in perturbed lakes in 2009 and in unperturbed lakes in 2008 and 2010, indicating a diet shift related to size. Brook trout became ^15^N-enriched with increasing body length, indicating an ontogenetic dietary shift.

### C Sources Supporting Food Webs

In oligotrophic lakes, like ours, humic matter originating from the catchment can be an important contribution of allochthonous matter for aquatic organisms [7], [8]. Furthermore, the loading of allochthonous matter can greatly exceed autochthonous primary production [38]. Subsidization from terrestrial habitats (leaf litter) appeared to be the main source of contribution for benthic primary consumers. Forest harvesting did not have an impact on the diet of the benthic animals, since leaf litter was the major contributor of organic matter in all treatments and years. This might not be surprising, as a buffer zone of 20 m around the watershed was left during logging.

### Brook Trout Diet-breath

Mixing models revealed that brook trout fed primarily on benthic predatory macroinvertebrates in all lakes, excepted in perturbed lakes in 2009. In this case, zooplankton was the main carbon source for brook trout with a contribution of 59%, indicating a change in brook trout diet in 2009 towards planktivory after forestry operations. Zooplankton also contributed 43% and 17% to brook trout diet in unperturbed lakes in 2009 and 2010 respectively, but predatory macroinvertebrates were still their major source of energy in both cases. The higher proportion of zooplankton observed in perturbed lakes fish's diet in 2009 could be explained by a higher abundance of zooplankton in perturbed lakes. In fact, in other studies in the same area, it has been shown that lakes affected by forest harvesting had higher *Daphnia* spp. abundance than unperturbed lakes [15], [39]. On the other hand, DOC concentrations have increased significantly in perturbed lakes in 2009. In 2010, DOC concentrations returned to their original condition as in 2008, suggesting that the system responded immediately after the perturbation and stabilized with time. Several studies have reported that forest harvesting in lake catchments can modify water quality, especially by producing higher DOC concentrations [14], [15], [39]. Since humic compounds in DOC constitute the major factor controlling water color in Canadian Boreal Shield lakes [40], the observed higher DOC concentrations in perturbed lakes may have provided a darker-colored environment in which zooplankton were more conspicuous for visual predators, such as brook trout. Brook trout, as other salmonids, is a visual predator and is known to feed on the most abundant and visible food items [41]. The ability of visually foraging fish to detect prey depends on light intensity, water clarity and prey characteristics [42]. Lower light intensity in perturbed lakes in 2009 can explain the higher proportion of zooplankton consumption by brook trout. Prey color and size have also a significant influence on brook trout prey detection. Benthic invertebrates are dark and become more cryptic in dark waters, compared to zooplankton which consistently offers greater contrast with their background environment [43]. Thus, brook trout likely fed more on zooplankton in 2009 because zooplankton abundance might be higher and because it might be easier to feed on them. In 2010, DOC levels returned to their original concentrations before the perturbation, increasing water transparency in waters and enabling brook trout to return to its original diet breath with predatory macroinvertebrates being the principal source contributor.

No logging impact was found on brook trout carbon and nitrogen isotopic values, since no significant differences were found for the interaction between treatment and year (Table 4) even if brook trout may have fed mainly on zooplankton in 2009, as revealed by SIAR model. However, δ^13^C and δ^15^N values for zooplankton were higher in 2009 and 2010 than in 2008 in both perturbed and unperturbed lakes, which may have masked any effect on brook trout isotopic values.

### Size-related Diet Shifts of Brook Trout

Brook trout became significantly more ^13^C-depleted with increasing fish total length in perturbed lakes in 2009 especially for the larger individuals and in unperturbed lakes in 2010, indicating a diet shift related to size. In small lakes, such as those sampled in our study, depletion in ^13^C with increasing length is generally indicative of a shift towards pelagic feeding [44]. This is consistent with mixing models results in 2009, where zooplankton was the principal carbon source. Brook trout largest individuals from perturbed lakes in 2009 could feed more in the pelagic zone as demonstrated by a highly depleted carbon stable isotope which is characteristic of pelagic consumers [8]. Conversely, fish from unperturbed lakes seemed to be associated with littoral and benthic zones. These results support our hypothesis that the decreased light intensity in perturbed lakes favours the detection of zooplanktonic preys by brook trout [23], [43].

δ^15^N ratios of brook trout became more positive with increasing fish total length in all lakes. This is typical of an ontogenetic dietary shift [22]. As fish increase in length and age, they are capable of handling larger prey items and ingest later instars of macroinvertebrates and terrestrial insects that fall into the lake. This has also been shown in other studies for brook trout [22], [23] and other fish species [45], [46]. There was no impact of logging on brook trout trophic level.

In conclusion, leaf-litter was the principal carbon source for benthic primary consumers in both perturbed and unperturbed lakes, showing no impact of forest harvesting on their diet. On the other hand, brook trout diet composition was affected by logging with greater zooplankton contribution possibly induced by lower light intensity in perturbed lakes or by a higher abundance of zooplankton in perturbed lakes in 2009. Future work will be needed to elucidate the mechanisms by which logging and increased DOC induces changes in brook trout diet. This change in brook trout diet was however mitigated in 2010, two years after the perturbation. Overall, forest harvesting seemed to have little short-term effect on trophic structure of eastern Canadian Boreal Shield lakes. Logging strategies used in this study such as CLAAG along with a 20-m buffer strip of standing forest around lakes might be an efficient protection to mitigate short-term effects of harvesting activities on watersheds on the Boreal Forest systems and should therefore not be underestimated. Finally, it has to be kept in mind that the period of this study of only one year before and two year after the perturbation is very short. Further long-term studies should significantly contribute to our understanding of how boreal lakes respond to disturbances to forest watersheds by forest management activities.
